# Acute respiratory distress syndrome in patients with hematological malignancies: a one-year retrospective nationwide cohort study

**DOI:** 10.1186/s13613-024-01373-4

**Published:** 2024-09-11

**Authors:** Pierre-Nicolas Bris, Vanessa Pauly, Véronica Orleans, Jean-Marie Forel, Pascal Auquier, Laurent Papazian, Laurent Boyer, Sami Hraiech

**Affiliations:** 1 Service de Médecine Intensive - Réanimation, AP-HM, Hôpital Nord, Marseille, France; 2 Faculté de médecine, Centre d’Etudes et de Recherches sur les Services de Santé et qualité de vie EA 3279, Aix-Marseille Université, Marseille, 13005 France; 3 Départment d’Informatique Médical, AP-HM, Marseille, France; 4 Centre Hospitalier de Bastia, Bastia, 20600 Corsica France

**Keywords:** Acute respiratory distress syndrome, Hematological malignancies, Mortality, Intensive care unit

## Abstract

**Background:**

Acute respiratory distress syndrome (ARDS) occurring in patients with hematological malignancies (HM) is a life-threatening condition with specific features. Mortality rate remains high but improvement has been described over the past several years. We aimed to describe characteristics and outcomes of ARDS in HM patients admitted in French ICUs (Intensive Care Units) during a one year-period. Data for this nationwide cohort study were collected from the French national hospital database (Programme de Médicalisation des Systèmes d’Information (PMSI)). All patients (18 years or older) admitted to French ICUs in 2017 and with a diagnosis of ARDS were included. Three groups were compared according to the presence of an HM, a solid cancer or no cancer. The primary endpoint was 90-day mortality. Secondary endpoints were the description of ICU management, etiologies of ARDS and mortality risk factors.

**Results:**

A total of 12 846 patients with ARDS were included. Among them, 990 had HM and 2744 had a solid cancer. The main malignancies were non-Hodgkin lymphoma (NHL) (28.5%), acute myeloid leukemia (AML) (20.4%) and multiple myeloma (19.7%). Day-90 mortality in patients with HM was higher than in patients with no cancer (64.4% vs. 46.6% *p* = 0.01) but was not different from that of patients with solid cancer (64.4% vs. 61.4%,*p* = 0.09). Intubation rate was lower in patients with HM in comparison with both groups (87.7% vs. 90.4% *p* = 0.02 for patients with solid cancer and 87.7% vs. 91.3%; *p* < 0.01 with no cancer). Independent predictors of mortality for patients with HM were a diagnosis of lymphoma or acute leukemia, age, a high modified SAPS II score, a renal replacement therapy, invasive fungal infection, and a septic shock. Bacterial pneumonia, extrapulmonary infections and non-invasive ventilation were protective.

**Conclusion:**

Mortality remains high in patients with HM admitted in ICU with ARDS in comparison with patients without cancer. Mortality predictors for this population were a diagnosis of lymphoma or acute leukemia, age, a high modified SAPS II score, a renal replacement therapy, invasive fungal infection and a septic shock.

**Supplementary Information:**

The online version contains supplementary material available at 10.1186/s13613-024-01373-4.

## Background

In the recent years, intensive care unit (ICU) admissions of patients with hematological malignancies (HM) have increased [1]. Among these patients, acute respiratory failure (ARF) is a leading cause. Acute respiratory distress syndrome (ARDS) is described in about 40% of HM patients admitted for ARF [2].

Because of specific features, patients with HM were often excluded from major ARDS studies [3, 4]. A few studies have therefore focused on ARDS in this specific population [5–7] showing a worst outcome than in the general population. Seong et al. [5] reported an ICU mortality around 57% in patients with HM admitted for ARDS while hospital mortality reached 64% in the cohort from Azoulay et al. [6]. As a comparison, in the Lung SAFE international report [8], hospital mortality was about 40% for ARDS patients in the overall population. Etiologies for ARDS are also specific in HM patients: they can be linked to immunodepression such as pulmonary aspergillosis or pneumocystosis [6] or directly related to the underlying malignancy: leukostasis [9], lymphangitis [10] or adverse events of oncological treatment [11]. Multiple factors can interact with ARDS evolution: disease status [6], type of malignancy [6], presence of neutropenia [2] or allogeneic hematopoietic stem cell transplantation (allo-HSCT) [12].

However, the data published until today included few patients, HM being rather rare diseases. This makes it particularly difficult to interpret the interaction between ARDS and a specific type of HM. Moreover, the largest series mostly included patients treated before 2010 and up to 1991 [6] whereas HM and ARDS management has considerably evolved over the past 30 years [13–15]. Finally, these cohorts often mixed patients with a diagnosis of HM and solid cancer although immunosuppression is deeper and more prolonged in patients with HM.

To update this data, we conducted a one-year nationwide study to compare ARDS mortality in patients with HM to patients with solid cancer or no cancer. Our secondary objectives were the comparison of ARDS etiology, severity, invasive support and duration of ICU stay in the same populations. We also tried to identify specific risk factors for mortality associated with the different subtypes of HM.

## Methods

### Data source

Data were collected from the French national hospital database (Programme de Médicalisation des Systèmes d’Information (PMSI)). PMSI systematically collects administrative and medical information related to all patients hospitalized and for every French hospital. The PMSI is based on diagnosis-related groups coded according to the International Classification of Diseases, Tenth Revision (ICD-10 French version) [16]. This database is accessible for researchers and health institutions who are collecting their data according to their commitment to respect guidelines. Research on such observational, retrospective and anonymous data are excluded from the framework of the French Law Number 2012 − 300 of the 5th of March 2012 relating to research involving human participants, as modified by the Order Number 2016 − 800 on the 16 th of June 2016. Approval of French competent authority (Agence Nationale de Sécurité du Médicament et des Produits de Santé, ANSM) or French ethics committee (Comité de Protection des Personnes, CPP) were not required. In accordance with the previous declaration of compliance with the reference methodology (MR005 N°: 2203797), the study was declared for ethical considerations to the French National Data Protection Commission.

### Study population

Patient-level data were obtained from the PMSI database for all patients admitted to an ICU from the 1st of January 2017, through the 31st of December 2017.

The inclusion criteria were as follows:

- age ≥ 18 years.
- ARDS code (J80) either as a primary diagnosis or occurring during the ICU stay.

Inside this population, we identified the patients with any type of cancer using the algorithm provided by the French national institute specialized in cancer (Institut National du Cancer (INCa)) [16]. We applied this algorithm to the ICU stay and to each hospital stay occurring in the 12 months before the ICU stay to define a “cancer patients base”. Within this cancer base, we identified patients either with a hematological malignancy (HM) or with a solid cancer (detailed codes for HM are listed in appendix in Table S1). Three groups were identified through this process:

- ARDS in patients with HM.
- ARDS in patients with solid cancer.
- ARDS in patients with no cancer.

A patient with both diagnoses (HM and solid cancer) was included in the HM group.

### Data collected

Patient characteristics included age, sex, Simplified Acute Physiology Score (SAPS) II score on admission and modified SAPS II (without the points from the underlying malignancy). The type of hospital (academic, other public hospitals, cancer hospitals or private) and the need for a re-hospitalization during the 12 months following ARDS were also considered. Comorbidities defined by the Charlson score were collected [17] and we computed the Charlson modified comorbidity index measuring the burden of disease by weighting these different comorbid conditions and by excluding the presence of an underlying malignancy from the score.

Type of hematological malignancy was determined using ICD-10 codes and was first classified in 11 categories: acute myeloid leukemia (AML), acute lymphoid leukemia (ALL), unknown type of acute leukemia, chronic lymphoid leukemia (CLL), multiple myeloma (MM), non-Hodgkin’s lymphoma (NHL), Hodgkin’s lymphoma (HL), myeloproliferative disorder (MPD) (divided into chronic myeloid leukemia, polycythemia vera, essential thrombocytemia, myelofibrosis and other type), myelodysplastic syndrome (MDS), chronic myelomonocytic leukemia (CMML) and other malignancy if patient was not classified elsewhere. For more clarity, we secondarily pooled these 11 categories into five: acute leukemia (gathering AML, ALL and unknown acute leukemia), lymphoma (gathering NHL and HL), MM, CLL and myelodysplastic syndrome/myeloproliferative disorder (gathering MDS, MPD and CMML). As one patient could be classified into several categories, we considered only the most clinically relevant, according to its impact on the patient’s prognosis. We considered acute leukemia as the worst prognosis followed by lymphoma then MM, MPD/MDS and finally CLL.

Oncological characteristics included:

- the presence of allogeneic and autologous Hematopoietic stem-cell transplantation (HSCT) during the ICU stay or up to 5 years before ARDS and delay between HSCT and ARDS.
- the presence of a chemotherapy session during the ICU stay or during the 12 months preceding the ICU stay and the delay between ARDS and the last session.
- the occurrence of grade 4 neutropenia during ICU stay.
- the need of red blood cell transfusion or other type of transfusion during the ICU stay.

We also collected oncological complications during the ICU stay:

- tumor lysis syndrome.
- disseminated intravascular coagulation (DIC),
- Graft-versus-host (GVH) disease,
- bronchial compression,
- adverse events of oncological treatments.
- leukostasis.

Detailed codes for oncological characteristics are listed in appendix 2.

ICU data collected were the need and duration for invasive mechanical ventilation (IMV), non-invasive ventilation (NIV), high-flow nasal cannula (HFNC) oxygen therapy, prone positioning, extracorporeal life support (ECLS) (including extracorporeal membrane oxygenation (venovenous or venoarterial) and extracorporeal carbon dioxide removal techniques), renal replacement therapy (RRT), the use of catecholamines, the presence of septic shock, surgery, a decision of withholding or withdrawing life-sustaining treatments (LST), duration of ICU and hospital stay as well as ICU, in-hospital and 90-day mortality (day-1 being the ICU admission of the index stay). We also collected 90-day mortality without including patients with a decision of withholding or withdrawing LST. Direct ICU admissions (ICU admission directly from home or emergency department without previous ward stay) were also reported.

Detailed codes for ICU data are listed in appendix 3.

Reason for ARDS were reported and classified as infection (pneumonia or extra-pulmonary sepsis), aspiration pneumonia, acute pancreatitis (AP) and trauma. We also constituted a group called “invasive fungal infections” (IFI) by combining pneumocystosis, pulmonary aspergillosis and other fungal pneumonia.

These different codes for ARDS etiologies were not mutually exclusive.

Codes for ARDS etiology are listed in appendix 4.

### Statistical methods

Characteristics and outcomes of ARDS patients with HM were compared to those in ARDS patients with no cancer and those in ARDS patients with solid cancer. Continuous variables were described as the mean ± standard deviation (SD) or median [interquartile range] and compared using Student’s t-test. Qualitative variables were described as counts (%) and compared using Chi-square test. Statistical significance was defined by a *p*-value < 0.05. Standardized difference was also calculated and was considered relevant when > 0.1 [18].

To provide further information on the prognostic factors of death at day 90 in overall population, a multilevel mixed-effects logistic model was generated with hospital as a random effect to account for correlation of the data at the hospital level. Center effect was evaluated with Intraclass correlation coefficient. We performed univariate and multivariable analyses. Variables in the univariate analysis with *p* < 0.20 and clinically relevant were introduced in the multivariable regression model. A backward selection method was performed with an alpha significance level of 0.05 (both for entry and retention). The results are shown as odds ratios (ORs) with 95% confidence intervals (CIs). Same analyses were performed in patients with HM only to identify more specific risk factors. For all statistical analyses, we used SAS Enterprise Guide version v 7.12.

## Results

**Fig. 1:**
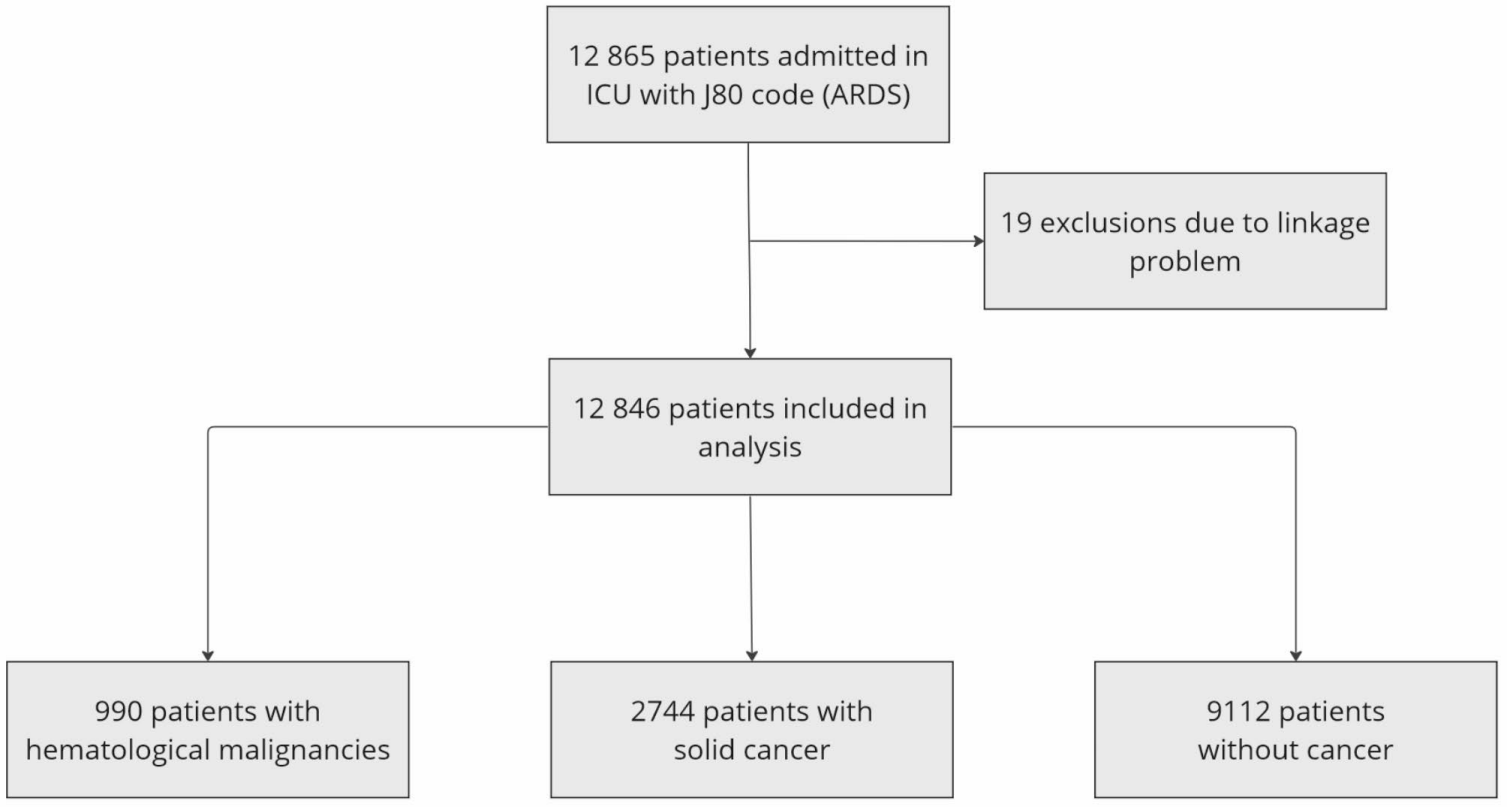
Study flowchart ICU: intensive care unit; ARDS: Acute respiratory distress syndrome

Figure 1 represents the study flowchart. During this one-year study period, 12,865 ICU patients had a diagnosis of ARDS. Among them, 990 (7.7%) were diagnosed with HM and 2744 (21.4%) with solid cancer. The main hematological malignancies were non-Hodgkin lymphoma (NHL) (28.5%), acute myeloid leukemia (AML) (20.4%) and multiple myeloma (19.7%). At ICU admission, SAPS II was higher in patients without cancer as compared with HM patients (42.71 +/- 23.35 vs. 39.57 +/- 24.11, *p* < 0.01) whereas there was no significant difference between HM and solid cancer patients (39.17 +/- 24.16 for cancer group, *p* = 0.66).

Patients’ characteristics and comparisons across groups are presented in Table 1. Detailed malignancies are listed in Table 2.

**Table 1:** Characteristics and comparison of ARDS patients

	Hematological Malignancies (*n* = 990)	Solid cancer (*n* = 2744)		No cancer (*n* = 9112)	HM vs. no cancer comparison		HM vs. solid cancer comparison		
					*p* value	Standardized difference	*p* value	Standardized difference	
**General characteristics**									
Age (years), mean (SD)	64.3 (14.1)	66.1 (11.6)		61.6 (16.6)	< 0.0001	**0.1769**	0.0005	**0.1344**	
Men	632 (63.84%)	1938 (70.63%)		6121 (67.18%)	0.0342	0.0702	0.0001	**0.1450**	
Charlson score without cancer > 3	334 (33.74%)	883 (32.18%)		3500 (38.41%)	0.0105	0.0974	0.5952	0.0332	
**Hospital characteristics**					< 0.0001	**0.3383**	< 0.0001	**0.3024**	
Public	316 (31.92%)	891 (32.47%)		3498 (38.39%)					
Academic	565 (57.07%)	1339 (48.80%)		4451 (48.85%)					
Cancer institute	25 (2.53%)	58 (2.11%)		18 (0.20%)					
Private	84 (8.50%)	456 (16.60%)		1145 (12.57%)					
**Hematological data**									
History of allo-HSCT	77 (7.78%)	0		0					
Allo-HSCT during ARDS stay	4 (0.40%)								
Delay between allo-HSCT and ARDS (days)	119.0 [36.00-363.0]								
History of auto-HSCT	51 (5.15%)	4 (0.15%)		2 (0.02%)	< 0.0001	**0.3274**	< 0.0001	0.3156	
Auto-HSCT during ARDS stay	2 (0.20%)	0		0	0.0096	0.0636	0.0702	0.0636	
Delay between auto-HSCT and ARDS (days)	135.0 [9.00-773.0]	787.5 [81.50–1502]		1267 [1112–1421]	< 0.0001	**0.1954**	< 0.0001	**0.1809**	
Chemotherapy the year before ARDS	482 (48.69%)	874 (31.85%)		1 (0.01%)	< 0.0001	**1.3769**	< 0.0001	**0.3484**	
Chemotherapy during ARDS stay	203 (20.51%)	124 (4.52%)		0	< 0.0001	**0.7183**	< 0.0001	**0.4979**	
Delay between chemotherapy and ARDS (days)	5.00 [0.00–33.00]	24.00 [7.00–67.00]		134			< 0.0001	**0.2572**	
Neutrophil count < 0.5 G/L	331 (33.43%)	211 (7.69%)		166 (1.82%)	< 0.0001	**0.9117**	< 0.0001	**0.6720**	
At least one red cell transfusion	490 (49.49%)	995 (36.26%)		2953 (32.41%)	< 0.0001	**0.3528**	< 0.0001	**0.2698**	
At least one other transfusion	343 (34.65%)	413 (15.05%)		1473 (16.17%)	< 0.0001	**0.4344**	< 0.0001	**0.4656**	

ARDS: acute respiratory distress syndrome HM: hematologic malignancies

HSCT: hematopoietic stem cell transplantation SD: standard deviation SAPS: Simplified Acute Physiology Score

**Table 2:** Detailed malignancies for patients with HM (*n* = 990)

Type of malignancy	*N*	%
**Acute leukemia**	243	24.6
Acute myeloid leukemia	202	20.4
Acute lymphoid leukemia	50	5.05
Acute leukemia of unknown type	26	2.63
**Myelodysplastic syndrome**	132	13.3
**Myeloproliferative disorder**	148	14.9
Polycythemia vera	33	3.3
Chronic myeloid leukemia	35	3.5
Essential thrombocytemia	43	4.3
Myelofibrosis	19	1.9
Other type of myeloproliferative disorder	43	4.3
**Chronic myelomonocytic leukemia**	26	2.6
**Lymphoma**	306	30.9
Hodgkin’s lymphoma	42	4.2
Non-Hodgkin’s lymphoma	282	28.5
**Multiple myeloma**	195	19.7
**Chronic lymphoid leukemia**	83	8.4
**Other type of malignancy**	193	19.5

### Outcomes (Table 3)

**Table 3:** Outcomes

	Hematological Malignancies (*n* = 990)	Solid cancer (*n* = 2744)	No cancer (*n* = 9112)	HM vs. no cancer comparison		HM vs. solid cancer comparison	
				*p* value	Standardized difference	*p* value	Standardized difference
**Outcomes**							
ICU mortality	595 (60.10%)	1570 (57.22%)	4046 (44.40%)	< 0.0001	**0.3182**	0.1149	0.0586
In-hospital mortality	627 (63.33%)	1661 (60.53%)	4273 (46.89%)	< 0.0001	**0.3351**	0.1209	0.0577
90-day mortality	638 (64.44%)	1685 (61.41%)	4249 (46.63%)	< 0.0001	**0.3644**	0.0910	0.0629
**Duration of stay**							
Duration of ICU stay, median (IQR), days	11.00 [3.00–20.00]	11.00 [5.00–22.00]	13.00 [5.00–26.00]	< 0.0001	**0.2100**	0.0395	0.0739
Duration of hospital stay, median (IQR), days	25.00 [11.00–44.00]	23.00 [11.00–42.00]	23.00 [10.00–43.00]	0.7121	0.0114	0.1970	0.0473

HM: hematological malignancies ICU: intensive care unit IQR: interquartile range

Day-90 mortality was higher for patients with HM (64.44%) as compared with patients with no cancer (46.63%, *p* < 0.01) but not as compared with patients with solid cancer (61.41%, *p* = 0.11). When patients with a decision of withholding or withdrawing LST were excluded, D-90 mortality became significantly higher in patients with HM (62.46%) as compared with solid cancer patients (58.40% *p* = 0.03).

### ICU management according to HM, solid cancer or no cancer status (Table 4)

**Table 4:** ICU management and oncological complications

	Hematological Malignancies (*n* = 990)	Solid cancer (*n* = 2744)	No cancer (*n* = 9112)	HM vs. no cancer comparison		HM vs. solid cancer comparison	
				*p* value	Standardized difference	*p* value	Standardized difference
**Oncological complications**	254 (25.66%)	257 (9.37%)	620 (6.80%)	0	**0.5289**	0	0.4388
Tumor lysis syndrome	67 (6.77%)	7 (0.26%)	0	< 0.0001	**0.3810**	< 0.0001	**0.3595**
Disseminated intravascular coagulation	91 (9.19%)	122 (4.45%)	557 (6.11%)	0.0002	**0.1160**	< 0.0001	**0.1891**
Graft-versus-host disease	41 (4.14%)	1 (0.04%)	0	< 0.0001	**0.2940**	< 0.0001	**0.2900**
Bronchial compression	5 (0.51%)	15 (0.55%)	15 (0.16%)	0.0399	0.0590	0.8778	0.0058
Adverse events of oncological treatments	106 (10.71%)	109 (3.97%)	33 (0.36%)	< 0.0001	**0.4645**	< 0.0001	**0.2604**
Leukostasis	6 (0.61%)	1 (0.04%)	5 (0.05%)	0.0003	0.0962	0.0019	**0.1008**
**ICU data**							
Modified SAPS II score at ICU admission, mean (SD)	39.57 (24.11)	39.17 (24.16)	42.71 (23.35)	< 0.0001	**0.1303**	0.6553	0.0166
Direct ICU admission	157 (15.86%)	457 (16.65%)	3053 (33.51%)	< 0.0001	**0.4181**	0.5625	0.0216
Invasive mechanical ventilation	868 (87.68%)	2480 (90.38%)	8317 (91.28%)	0.0002	**0.1175**	0.0167	0.0865
Duration of invasive mechanical ventilation, median (days)	8.00 [3.00–17.00]	9.00 [3.00–18.00]	11.00 [4.00–22.00]	< 0.0001	**0.2006**	0.0644	0.0698
Non invasive ventilation	363 (36.67%)	1010 (36.81%)	3180 (34.90%)	0.2683	0.0369	0.9372	0.0029
Duration of non-invasive ventilation, median (days)	2.00 [1.00–5.00]	3.00 [1.00–5.00]	3.00 [1.00–6.00]	0.2992	0.0549	0.9793	0.0017
High flow nasal canula	205 (20.71%)	477 (17.38%)	1356 (14.88%)	< 0.0001	**0.1528**	0.0203	0.0847
Duration of high flow nasal cannula, median (days)	2.00 [1.00–4.00]	3.00 [1.00–5.00]	3.00 [1.00–5.00]	0.0462	**0.1313**	0.0137	**0.1930**
Use of vasopressors	814 (82.22%)	2217 (80.79%)	7392 (81.12%)	0.4005	0.0284	0.3246	0.0368
Prone positioning	249 (25.15%)	649 (23.65%)	2508 (27.52%)	0.1115	0.0539	0.3438	0.0349
ECLS	36 (3.64%)	79 (2.88%)	565 (6.20%)	0.0012	**0.1188**	0.2371	0.0427
Duration of ECLS, median (days)	4.00 [1.00- 9.50]	5.00 [1.00–11.00]	5.00 [2.00–10.00]	0.7138	0.0649	0.6897	0.0753
Surgical procedure	379 (38.28%)	1560 (56.85%)	3906 (42.87%)	0.0056	0.0934	< 0.0001	**0.3784**
Renal replacement therapy	369 (37.27%)	775 (28.24%)	2824 (30.99%)	0.0001	**0.1328**	< 0.0001	**0.1933**
Decision of withholding or withdrawing LST	103 (10.40%)	333 (12.14%)	409 (4.49%)	< 0.0001	**0.2268**	0.1459	0.0548
Septic shock	583 (58.89%)	1507 (54.92%)	4206 (46.16%)	< 0.0001	**0.2570**	0.0310	0.0802

ECLS: extracorporeal life support ICU: intensive care unit LST: life-sustaining treatments

Intubation rate was significantly lower for HM patients (87.68% vs. 90.38%, *p* = 0.02 for cancer patients and 91.28%, *p* < 0.01 for no cancer patients). HFNC oxygen therapy rate was higher in HM patients (20.71% vs. 17.38%, *p* = 0.02 for cancer patients and 14.88%, *p* < 0.01 for no cancer group). RRT was more frequently used (37.27%) in comparison with patients with solid cancer (28.24% *p* < 0.01) or without cancer (30.99% *p* < 0.01). ECLS was found for 36 patients with HM (3.64%), which is significantly lower than patients with no cancer (6.20%, *p* < 0.01).

### HM vs. solid cancer and no cancer patients’ ARDS etiology comparison (Table 5)

**Table 5:** ARDS etiology and associated infections

	Hematological Malignancies (*n* = 990)	Solid cancer (*n* = 2744)	No cancer (*n* = 9112)	HM vs. no cancer comparison			HM vs. solid cancer comparison		
				*p* value		Standardized difference	*p* value		Standardized difference
**Pneumonia**	692 (69.90%)	1644 (59.91%)	5614 (61.61%)	< 0.0001		**0.1753**	< 0.0001		**0.2104**
Bacterial pneumonia	502 (50.71%)	1291 (47.05%)	4614 (50.64%)	0.9664		0.0014	0.0482		0.0732
Viral pneumonia	80 (8.08%)	83 (3.02%)	485 (5.32%)	0.0003		**0.1105**	< 0.0001		**0.2221**
Pneumocystosis	65 (6.57%)	72 (2.62%)	98 (1.08%)	< 0.0001		**0.2894**	< 0.0001		**0.1891**
Pulmonary aspergillosis	45 (4.55%)	33 (1.20%)	102 (1.12%)	< 0.0001		**0.2076**	< 0.0001		**0.2011**
Other fungal pneumonia	34 (3.43%)	43 (1.57%)	103 (1.13%)	< 0.0001		**0.1547**	0.0004		**0.1198**
Invasive fungal infections	109 (11.01%)	100 (3.64%)	196 (2.15%)	< 0.0001		**0**,**3631**	< 0.0001		**0**,**2855**
Undocumented pneumonia	247 (24.95%)	559 (20.37%)	1581 (17.35%)	< 0.0001		**0.1869**	0.0027		**0.1095**
**Extrapulmonary infections**	423 (42.73%)	1186 (43.22%)	3402 (37.34%)	0.0009		**0.1102**	0.7877		0.0100
Urinary tract infection	82 (8.28%)	287 (10.46%)	929 (10.20%)	0.0569		0.0661	0.0492		0.0747
Abdominal sepsis	126 (12.73%)	438 (15.96%)	982 (10.78%)	0.0622		0.0606	0.0148		0.0924
Cutaneous sepsis	98 (9.90%)	266 (9.69%)	698 (7.66%)	0.0130		0.0792	0.8520		0.0069
Bacteriemia / candidemia	294 (29.70%)	745 (27.15%)	2120 (23.27%)	< 0.0001		**0.1461**	0.1253		0.0565
**Trauma**	91 (9.19%)	365 (13.30%)	1928 (21.16%)	< 0.0001		**0.3383**	0.0007		**0.1304**
**Acute pancreatitis**	10 (1.01%)	53 (1.93%)	438 (4.81%)	< 0.0001		**0.2274**	0.0537		0.0766
**Aspiration pneumonia**	100 (10.10%)	552 (20.12%)	2038 (22.37%)	< 0.0001		**0.3373**	< 0.0001		**0.2824**

Total is greater than 100% because patients could have more than 1 associated infection

Pneumonia was the main ARDS etiology in HM patients (69.90% vs. 59.91%, *p* < 0.01 for cancer patients and 61.61%, *p* < 0.01 for no cancer patients). Infectious agents typically associated with immunocompromised patients such as viruses, pneumocystis and aspergillus were more frequently found in HM patients. Extrapulmonary infections were more frequent in HM patients (42.73%) as compared with patients with no cancer (37.34%, *p* < 0.01) but not with cancer patients (43.22%, *p* = 0.79).

Main etiologies of extrapulmonary sepsis among HM patients were bacteremia (29.70%), abdominal sepsis (12.73%), cutaneous sepsis (9.90%) and urinary tract infections (8.28%).

### Mortality risk factors in HM patients (Tables 6 and 7)

**Table 6:** Factors associated with 90-day mortality in overall population

Variable	Univariate analysis			Multivariate analysis		
	Odds ratio	95% CI	*P* value	Adjusted Odds ratio	Adjusted 95% CI	Adjusted *P* value
**Modified SAPS II (without age)**	1.023	1.021–1.025	< 0.0001	1.019	1.018–1.021	< 0.0001
**Age**	1.040	1.030–1.040	< 0.0001	1.042	1.039–1.045	< 0.0001
**Septic shock**	1.720	1.600–1.850	< 0.0001	1.192	1.097–1.296	< 0.0001
**Renal replacement therapy**	2.900	2.670–3.140	< 0.0001	2.456	2.241–2.691	< 0.0001
**Group**						
No cancer	**1**	**Reference**		**1**	**Reference**	
Solid cancer	1.840	1.680–2.000	< 0.0001	1.889	1.709–2.087	< 0.0001
HM	2.080	1.820–2.380	< 0.0001	2.219	1.900–2.591	< 0.0001
**Pneumonia**	0.580	0.540–0.620	< 0.0001	0.661	0.607–0.719	< 0.0001
**Trauma**	0.620	0.570–0.680	< 0.0001	0.735	0.662–0.816	< 0.0001
**Acute pancreatitis**	0.760	0.640–0.910	0.0034	0.731	0.592–0.903	0.0036
**High flow nasal canula**	0.510	0.460–0.570	< 0.0001	0.674	0.602–0.754	< 0.0001
**Non-invasive ventilation**	0.420	0.390–0.450	< 0.0001	0,472	0.432–0.514	< 0.0001
**Hospital characteristics**						
Private	**1**	**Reference**		**1**	**Reference**	
Public and cancer institute	1.130	0.950–1.340	0.1577	1.277	1.061–1.536	0.0097
Academic	1.020	0.840–1.240	0.8213	1.280	1.038–1.577	0.0212
**Mechanical ventilation**	1.640	1.450–1.860	< 0.0001			0.0982
**Aspiration pneumonia**	0.810	0.740–0.880	< 0.0001			0.6231
**Charlson score without cancer**	1.050	1.030–1.060	< 0.0001			0.4624
**Direct ICU admission**	0.910	0.841–0.985	0.0191			0.1990
**Extrapulmonary infections**	0.710	0.660–0.760	< 0.0001			0.7183

CI: confidence interval HM : hematological malignancies SAPS: Simplified Acute Physiology Score

Intraclass correlation coefficient: 2.2%

**Table 7:** Factors associated with 90-day mortality in patients with HM

Variable			Univariate analysis			Multivariate analysis			
			Odds ratio	95% CI	*P* value	Adjusted Odds ratio	Adjusted 95% CI	Adjusted *P* value	
**Modified SAPS II (without age)**			1.026	0.985–1.068	0.0786	1.023	1.016–1.03	< 0.0001	
**Age**			1.010	1.000–1.020	0.0080	1.030	1.018–1.041	< 0.0001	
**Type of malignancy**								0.0006	
Multiple myeloma			**1**	**Reference**		**1**	**Reference**		
CLL			1.290	0.680–2.430	0.4393	**1.090**	0.532–2.230	0.8143	
MPD / MDS			1.230	0.810–1.850	0.3309	**1.430**	0.898–2.278	0.1316	
Other type of malignancy			1.290	0.600–2.750	0.5101	**1.702**	0.721–4.017	0.2246	
Acute leukemia			1.720	1.150–2.580	0.0079	**2.410**	1.503–3.865	0.0003	
Lymphoma			1.890	1.280–2.790	0.0014	**2.422**	1.558–3.763	< 0.0001	
**Non-invasive ventilation**			0.520	0.400–0.680	< 0.0001	0.658	0.484–0.895	0.0076	
**Renal replacement therapy**			3.470	0.490–24.770	0.0788	3.293	2.351–4.612	< 0.0001	
**Septic shock**			1.970	0.350–10.930	0.1256	1.652	1.201–2.271	0.002	
**Bacterial pneumonia**			0.720	0.550–0.930	0.0136	0.721	0.533–0.975	0.0338	
**Extrapulmonary infections**			0.790	0.610–1.040	0.0884	0.565	0.415–0.771	0.0003	
**Invasive fungal infections**			1.599	1.020–2.507	0.0407	1.732	1.049–2.858	0.0317	
**Allo-HSCT**			1.240	0.740–2.050	0.4121	1.753	0.953–3.225	0.0711	
**Neutropenia**			1.340	1.010–1.780	0.0400			0.4289	
**Chemotherapy before ARDS**			1.180	0.910–1.530	0.2158			0.8023	
**Mechanical ventilation**			1.980	0.170–23.570	0.1765			0.4435	
**Direct ICU admission**			0.818	0.575–1.164	0.2643			0.1056	
**High flow nasal canula**			0.670	0.490–0.920	0.0145			0.6789	
**Viral pneumonia**			0.680	0.430–1.090	0.1117			0.4343	
**Hospital characteristics**								0.2698	
Private			**1**	**Reference**					
Public and cancer institute			2.510	0.040–147.160	0.2131				
Academic			2.660	0.050–144.790	0.1982				

ARDS: Acute respiratory distress syndrome CI: confidence interval SAPS: Simplified Acute Physiology Score CLL: Chronic lymphoid leukemia MPD: Myeloproliferative disorder MDS: Myelodysplastic syndrome HSCT: hematopoietic stem cell transplantation

Intraclass correlation coefficient: 0.23

Patients with acute leukemia (OR 2.41 (1.50–3.87), *p* < 0.01) and lymphoma (OR 2.42 (1.56–3.76), *p* < 0.01) had a higher risk for mortality in comparison with patients with MM. Other factors associated with a worse prognosis were age (OR 1.03 (1.02–1.04), *p* < 0.01), higher modified SAPS II at ICU admission (OR 1.02 (1.02–1.03), *p* < 0.01), septic shock (OR 1.65 (1.20–2.27, *p* < 0.01) and the need for renal replacement therapy (OR 3.29 (2.35–4.61), *p* < 0.01).

Concerning oxygen devices, NIV was protective (OR 0.66 (0.48–0.90); *p* < 0.01) whereas IMV and HFNC were not independently related to mortality (*p* = 0.44 and *p* = 0.68 respectively).

Among ARDS etiologies, bacterial pneumonia and extrapulmonary infections were associated with a lower mortality (OR 0.72 (0.53–0.98); *p* = 0.03 and 0.57 (0.42–0.77); *p* < 0.01 respectively) unlike invasive fungal infections (OR 1.73 (1.05–2.86); *p* = 0.03).

## Discussion

Our study aimed to characterize the patients diagnosed with HM and admitted to the ICU for ARDS. In 2017, 990 patients with a diagnosis of HM were admitted for ARDS in French ICUs, representing 7.7% of all ARDS cases. The main malignancies were NHL, AML and MM. Six hundred and thirty-eight (64.4%) patients died within the 90 days following admission for ARDS, which was significantly higher than for patients without cancer (46.6%).

Among the most recent studies in ARDS patients with HM, mortality varies from 57% [5] to 77% [7]. Azoulay [6] et al. described a 64% hospital mortality but noticed a significant reduction over time, dropping from 89% in 1990-95 to 52% in 2006-11. Our results confirmed a high mortality with a more recent cohort and patients older than in previous series. Moreover, we included all type of hospitals whereas previous cohorts focused on ICUs with a high experience in managing patients with ARDS and malignancies [6], though possibly overestimating survival. Of note, after exclusion of patients with withholding treatment decisions, HM patients had a higher mortality than solid cancer, mortality remaining high, comparably to what was previously described [6]. As end of life decisions are driven by various factors, this result must be interpreted with caution.

Interestingly, multiple myeloma was frequent (19.7%) in our study whereas previous series focusing on ARDS patients with HM [6] described a significant reduction over time (from 28 to 5%). Because ARDS patients with lymphoma or AML can represent more diagnostic and therapeutic challenges, they might have been more frequently hospitalized in highly specialized ICUs than other malignancies. This could have led to a selection bias in previous studies and caused an underestimation of MM occurrence.

Most of the previous studies reported about 30% of viral pneumonia, from 8 to 10% of pneumocystosis and about 20% of pulmonary aspergillosis in HM patients. These rates were lower in our study, which may be related to multiple factors. Previous studies were conducted in experienced ICUs with a greater proportion of allo-HSCT, AML or NHL patients and familiar to complex diagnostic strategies. Another hypothesis could be the decrease incidence of viral or fungal infections because of the better use of antiviral and anti-fungal prophylaxis for several malignancies [19, 20].

Intubation rate of HM patients in the study is in keeping with previous studies [21] and is lower than for patients with no cancer (87.7% vs. 91.3%). HFNC was quite frequent (20.7%), especially as compared with patients with no cancer (14.5%). This could be explained by the increased mortality described in immunocompromised patients under invasive mechanical ventilation [22]. ECLS concerned 3.6% of patients with HM, which is rather high in comparison with literature, given that HM are considered relative contraindications [23]. A recent South Korean study [24] reported a rate for ECLS in patients with ARDS ranging from 5 to 8%.

Previous determination of mortality predictors found invasive aspergillosis, allo-HSCT, neutropenia or a refractory disease for this specific population in addition to classical predictors [5, 6] in ARDS. In our study, mortality risk factors for HM patients were the presence of AML, NHL or IFI while neutropenia was not. Allo-HSCT was not independently related to mortality (*p* = 0.07) but this condition was little represented in our cohort (7.78%). We found that IMV was not related to mortality in HM patients, contrary to what was previously reported [6, 25]. This might reflect a better accordance with protective ventilation in the recent years. The large number of patients under IMV also suggests a careful selection of patients before intubation. Non-invasive ventilation appeared as a protective factor. HFNC was protective in the overall population but not in HM patients. However, as we couldn’t identify the time sequence of oxygen devices, it is difficult to draw conclusion on this point. The diagnosis of bacterial pneumonia also appeared to be protective. In HM patients, failure to diagnose ARDS etiology is probably associated with a worse prognosis, as already described [26].

Our study carries some limitations. First, diagnosis of ARDS was made by ICU clinicians without a priori defined criteria. Moreover, some patients with mild form of ARDS may have been admitted in intermediate care/step-down units and were not included. This could have led to an overestimation of mortality rate. Conversely, the exclusion of patients for whom admission to the ICU was disclaimed because of ethical considerations might have lowered mortality rates.

Data collected were only based on hospital records. Deaths occurring after hospital discharge were therefore not included. However, patients usually die during hospitalization, as previously described [27].

Finally, disease status was not known. Patients in complete remission may have been included, reducing the impact of these malignancies on ARDS. However, the rate of patients with severe neutropenia was rather high (33%) and transfusions were frequent, suggesting a high proportion of patients with active malignancy or recent treatment. Moreover, patients receiving chemotherapy the year before ARDS was not found as an independent predictor of death.

## Conclusions

Mortality in patients diagnosed with ARDS and HM remains high in comparison with ARDS patients with no cancer, especially with a diagnosis of AML or NHL. Moreover, patients were older than those in previous studies, suggesting less stringent criteria for ICU admission and therefore improvement in ARDS management of patients with HM.

## Electronic supplementary material

Below is the link to the electronic supplementary material.

Supplementary Material 1

## Data Availability

The datasets used and/or analyzed during the current study are available from the corresponding author on reasonable request.

## Abbreviations

ALL: Acute lymphoid leukemia
AML: Acute myeloid leukemia
ANSM: Agence Nationale de Sécurité du Médicament et des Produits de Santé
AP: Acute pancreatitis
ARDS: Acute respiratory distress syndrome
ARF: Acute respiratory failure
CIs: Confidence intervals
CLL: Chronic lymphoid leukemia
CMML: Chronic myelomonocytic leukemia
CPP: Comité de protection des personnes
DIC: Disseminated intravascular coagulation
ECLS: Extracorporeal life support
GVH: Graft-versus-host
HFNC: High-flow nasal cannula
HL: Hodgkin’s lymphoma
HM: Hematological malignancies
HSCT: Hematopoietic stem-cell transplantation
ICD-10: International Classification of Diseases, Tenth Revision
ICU: Intensive Care Unit
IFI: invasive fungal infections
IMV: Invasive mechanical ventilation
INCa: Institut National du Cancer
LST: Life-sustaining treatments
MM: Multiple myeloma
MDS: Myelodysplastic syndrome
MPD: Myeloproliferative disorder
NHL: Non-Hodgkin lymphoma
NIV: Non-invasive ventilation
ORs: Odds ratios
PMSI: Programme de Médicalisation des Systèmes d’Information
RRT: Renal replacement therapy
SAPS: Simplified Acute Physiology Score
SD: Standard deviation
