# Supplementary material for: Acute respiratory distress syndrome in patients with hematological malignancies: a one-year retrospective nationwide cohort study
Source: Ann Intensive Care. 2024 Sep 11;14:141. doi: 10.1186/s13613-024-01373-4 (PMC11390989; doi:10.1186/s13613-024-01373-4)
Supplement: Supplementary file 1 — Supplementary Material 1 [file 13613_2024_1373_MOESM1_ESM.docx]

Appendix

A/ Codes :

1. Hematological malignancies

| **Table S1 : Codes and classification of hematological malignancies** | | |
| --- | --- | --- |
| Acute myeloid leukemia | | C92.0, C92.3, C92.4, C92.5, C92.6, C92.8 C93.0, C94.0, C94.2, C94.4 |
| Acute lymphoid leukemia | | C83.5 C91.0 |
| Unknown type of acute leukemia | | C91.8  C95.0 |
| Myelodysplastic syndrome |  | D46.0 à D46.9 |
| Myeloproliferative disorder | Polycythemia vera | C94.0, C94.1  D45 |
|  | Chronic myeloid leukemia | C92.1, C92.2 |
|  | Essential thrombocytemia | D47.3 |
|  | Myelofibrosis | C94.5 D47.4 |
|  | Other type | C94.6  D47.1, D47.5 |
| Non-Hodgkin’s lymphoma | | B21.1, B21.2 C82.0 to C83.4 C83.6 to C86.6  C88.0, C88.3, C88.4 C96.3 |
| Hodgkin’s lymphoma | | C81.0 to C81.9 |
| Chronic lymphoid leukemia |  | C91.1, C91.4, C91.6 |
| Multiple myeloma |  | C90.0 C90.1 C90.2 C90.3 D47.2 |
| Chronic myelomonocytic leukemia |  | C93.1 |
| Other malignancy |  | If not already classified elsewhere : C81* to C96*  D46*, D47 |

1. Oncological characteristics

Allogeneic Hematopoietic stem-cell transplantation :
- ICD-10 codes : Z94802, Z94803, Z94804, Z94809
- CCAM) : FELF009

Autologous Hematopoietic stem-cell transplantation :
- ICD-10 codes : Z94800
- CCAM : FELF010

Chemotherapy :
- ICD-10 codes : Z51.1,Z51.2
- CCAM : AFLB003

Grade IV neutropenia :
- ICD-10 codes : D70, D611, D612, D618, D619

Red blood cell transfusion :
- CCAM : FELF004 FELF011, FELF001

Other type of transfusion :
- CCAM : FELF003, FELF006

Tumor lysis syndrome :
- ICD-10 code : E883

Disseminated intravascular coagulation :
- ICD-10 code : D65

Graft-versus-host (GVH) disease :
- ICD-10 code : T860

Bronchial compression :
- CCAM : GELE008, GENE001, GENE002, GENE003, GENE004, GENE006, GENE008, which are codes for bronchial compression treatment

Adverse events of oncological treatments :
- ICD-10 codes : Y430, Y431, Y432, Y433, Y434

Leukostasis :
- CCAM : FEPF 004 (which is the code for leukapheresis)

1. ICU therapies

Mechanical ventilation :
- CCAM: GLLD004, GLLD008, GLLD007, GLLD006, GLLD013, GLLD015

Non-invasive ventilation :
- CCAM: GLLD019, GLLD012

High-flow nasal cannula oxygen therapy :
- CCAM : GLLD003

Prone positioning :
- CCAM: GLLD004

Extracorporeal life support :
- CCAM: EQLA002, EQQP004, GLJF010

Renal replacement therapy :
- CCAM: JVJF005, JVJF002, JVJF003, JVJF004, JVJF008

Use of catecholamines :
- CCAM: EQLF003

Presence of septic shock :
- ICD-10 code : R572

Decision of withholding or withdrawing life-sustaining treatments:
- ICD-10 code: Z515

Surgery :
- ADC classification in CCAM

1. ARDS etiology

Bacterial pneumonia :
- ICD-10: A221, A481, J13, J14, J15.0, J151, J152, J153, J154, J155, J156, J157, J158, J159, J160, J170, A430, A492, A493, J852, J853, J86*)

Viral pneumonia :
- ICD-10 : B25.0, J10.0, J11.0, J12.0, J12.1, J12.2, J12.3, J12.8, J12.9, J17.1

Pneumocystosis :
- ICD-10 : B59

Fungal pneumonia :
- ICD-10 : B440, B441 for aspergillosis and B450, B460, J172, B380, B390, B400, B447, B457, B464, J173 for other fungal infections

Undocumented pneumonia :
- ICD-10 : J168, J178, J180, J181, J182, J188, J189

Urinary sepsis :
- ICD-10: N10, N390, T835

Abdominal sepsis :
- ICD-10: K750, K800, K804, K810, O85, K570, K572, K574, K578, A00*, A01*, A020, A03*, A04*, A07*, A08*, A09*, K35*, K650

Skin or catheter infection :
- ICD-10: A48.0, L02*, L03*, L080, M726, T827, T857

Isolated bacteremia or undetermined infection :
- ICD-10: A40*, A021, A227, A267, A327, A427, A410, A411, A412, A413, A414, A415, A498, A499, A483, A490, A491, B377, B581, B582, B588, B589, A438, A439

Aspiration pneumonia :
- ICD-10 code J69* and J68*

Acute pancreatitis :
- ICD-10 codes : K85* and B252

Trauma :
- ICD-10 codes : from S00* to T35*, from T66* to T71* and T79*.

B/ Subgroup analysis

| **Table S2 : Subgroup analysis** | | | | | | | | | | | | |
| --- | --- | --- | --- | --- | --- | --- | --- | --- | --- | --- | --- | --- |
|  | **Type of malignancy** | | | | | | | | | | | |
|  | **Acute leukemia, %**  **(n=243)** | | **Chronic lymphoid leukemia, %  (n=52)** | | **Lymphoma, %**  **(n=286)** | | **Multiple myeloma, %**  **(n=180)** | | **MDS/SMP, %**  **(n=195)** | | **Other, %**  **(n=34)** | |
| **90-day mortality** | 68,31 | | 61,54 | | 70,28 | | 55,56 | | 60,51 | | 61,76 | |
| **Decision of withholding LST** | 7,82 | | 7,69 | | 12,59 | | 11,67 | | 9,23 | | 14,71 | |
| **Age in years, median [IQR]** | 61 [23] | | 72 [13,5] | | 67 [15] | | 68 [17,5] | | 69 [19] | | 60 [22] | |
| **Pneumonia** | 68,31 | | 76,92 | | 72,03 | | 73,33 | | 64,10 | | 67,65 | |
| Bacterial pneumonia | 45,68 | | 53,85 | | 51,05 | | 57,22 | | 49,23 | | 52,94 | |
| Viral pneumonia | 6,58 | | 9,62 | | 9,09 | | 8,89 | | 6,67 | | 11,76 | |
| Pneumocystosis | 4,53 | | 11,54 | | 8,74 | | 5,56 | | 5,13 | | 8,82 | |
| Pulmonary aspergillosis | 5,76 | | 3,85 | | 5,94 | | 3,33 | | 2,05 | | 5,88 | |
| Other fungal pneumonia | 4,94 | | . | | 3,50 | | 3,89 | | 2,05 | | 2,94 | |
| Undocumented pneumonia | 27,57 | | 28,85 | | 24,83 | | 25,00 | | 22,05 | | 17,65 | |
| **Aspiration pneumonia** | 3,70 | | 3,85 | | 9,09 | | 14,44 | | 16,92 | | 11,76 | |
| **Extrapulmonary infections** | 46,91 | | 28,85 | | 45,80 | | 42,78 | | 35,90 | | 47,06 | |
| Urinary tract infection | 4,94 | | 9,62 | | 8,39 | | 11,11 | | 9,74 | | 5,88 | |
| Abdominal sepsis | 14,40 | | 7,69 | | 11,89 | | 11,11 | | 12,31 | | 26,47 | |
| Cutaneous sepsis | 14,81 | | 7,69 | | 9,79 | | 8,33 | | 7,18 | | 2,94 | |
| Bacteriemia / candidemia | 34,16 | | 19,23 | | 32,17 | | 30,00 | | 24,10 | | 23,53 | |
|  |  |  |  |  |  |  |  |  |  |  |  |  |
| **Oncological complications** | 46,09 | | 9,62 | | 26,57 | | 12,78 | | 13,85 | | 23,53 | |
| Tumor lysis syndrome | 15,23 | | 1,92 | | 8,39 | | 1,11 | | 1,54 | | . | |
| Disseminated intravascular coagulation | 17,28 | | 3,85 | | 8,04 | | 5,00 | | 6,15 | | 8,82 | |
| Graft-versus-host disease | 9,88 | | . | | 2,10 | | 2,22 | | 3,08 | | 2,94 | |
| Bronchial compression | . | | 1,92 | | 1,05 | | . | | 0,51 | | . | |
| Adverse events of treatments | 18,93 | | 3,85 | | 12,94 | | 4,44 | | 4,62 | | 11,76 | |
| Leukostasis | 2,06 | | . | | . | | . | | . | | 2,94 | |

*IQR: Interquartile range LST: life-sustaining treatments MPD : Myeloproliferative disorder MDS :* *Myelodysplastic syndrome*

C/ Univariate analysis in overall population

| **Table S3 : Factors associated with 90-day mortality in overall population** **(univariate analysis)** | | | |
| --- | --- | --- | --- |
| **Variable** | **Odds ratio** | **95% CI** | **P value** |
| **Sexe (Male vs Female)** | 1.03 | 0.96 – 1.12 | 0.3677 |
| **Neutropenia** | 2.02 | 1.72 – 2.38 | <0.0001 |
| **At least one red cell transfusion** | 0.99 | 0.92 – 1.07 | 0.8526 |
| **At least one other transfusion** | 1.73 | 1.57 – 1.91 | <0.0001 |
| **Prone position** | 0.93 | 0.86 – 1.01 | 0.0718 |
| **ECLS** | 1.23 | 1.05 – 1.44 | 0.0101 |
| **Use of vasopressors** | 2.56 | 2.32 – 2.81 | <0.0001 |
| **Surgical procedure** | 0.66 | 0.61 – 0.71 | <0.0001 |
| **Decision of withholding LST** | 5.38 | 4.47 – 6.48 | <0.0001 |
| **Bacterial pneumonia** | 0.58 | 0.54 – 0.62 | <0.0001 |
| **Viral pneumonia** | 0.69 | 0.59 – 0.81 | <0.0001 |
| **Pneumocystosis** | 1.17 | 0.68 – 2.01 | 0.5723 |
| **Aspergillosis** | 2.28 | 1.08 – 4.48.0 | 0.0303 |
| **Other fungal pneumonia** | 0.78 | 0.39 – 1.57 | 0.4892 |
| **Other type of pneumonia** | 0.84 | 0.77 – 0.92 | <0.0001 |
| **Urinary tract infection** | 0.51 | 0.45 – 0.57 | <0.0001 |
| **Abdominal sepsis** | 0.77 | 0.69 – 0.86 | <0.0001 |
| **Cutaneous sepsis** | 0.51 | 0.45 – 0.58 | <0.0001 |
| **Bacteriemia / candidemia** | 0.84 | 0.77 – 0.91 | <0.0001 |
| **Duration of invasive mechanical ventilation** | 0.97 | 0.94 – 0.99 | <0.0001 |
| **Duration of non-invasive ventilation** | 0.98 | 0.97 – 0.99 | 0.0002 |
| **Duration of high flow nasal cannula** | 0.97 | 0.94 – 0.99 | 0.0026 |
| *ARDS : acute respiratory distress syndrome CI : confidence interval ECLS : extracorporeal life support LST : life-sustaining treatments* | | | |

D/ Univariate analysis in patients with hematological malignancies

| **Table S4 : Factors associated with 90-day mortality in HM population** **(univariate analysis)** | | | |
| --- | --- | --- | --- |
| **Variable** | **Odds ratio** | **95% CI** | **P value** |
| **Sexe (Male vs Female)** | 1.01 | 0.77 – 1.33 | 0.9209 |
| **At least one red cell transfusion** | 1.04 | 0.80 – 1.35 | 0.7778 |
| **At least one other transfusion** | 1.72 | 0.27 – 10.85 | 0.1662 |
| **Prone position** | 1.09 | 0.80 – 1.48 | 0.5794 |
| **ECLS** | 0.86 | 0.43 – 1.71 | 0.6717 |
| **Use of vasopressors** | 2.69 | 0.31 – 23.15 | 0.1078 |
| **Surgical procedure** | 0.59 | 0.45 - 0.77 | 0.0001 |
| **Decision of withholding LST** | 2.66 | 0.09 – 75.44 | 0.1675 |
| **Undocumented pneumonia** | 0.79 | 0.12 – 5.40 | 0.3629 |
| **Urinary tract infection** | 0.47 | 0.30 – 0.74 | 0.0011 |
| **Abdominal sepsis** | 0.81 | 0.55 – 1.19 | 0.2851 |
| **Cutaneous sepsis** | 0.67 | 0.44 – 1.03 | 0.0697 |
| **Bacteriemia / candidemia** | 1.05 | 0.79 – 1.41 | 0.7242 |
| **Tumor lysis syndrome** | 1.82 | 0.04 – 77.06 | 0.2900 |
| **Disseminated intravascular coagulation** | 2.58 | 1.50 – 4.446 | 0.0007 |
| **Graft-versus-host disease** | 0.85 | 0.44 – 1.63 | 0.6176 |
| **Bronchial compression** | 0.83 | 0.14 – 5.00 | 0.8346 |
| **Adverse events of oncological treatments** | 0.82 | 0.54 – 1.24 | 0.3478 |
| **Leukostasis** | 0.27 | 0.05 – 1.52 | 0.1382 |
| **Allo-HSCT during ICU stay** | 1.64 | 0.17 – 16.12 | 0.6703 |
| **Delay between allo-HSCT and ARDS** | 1.00 | 1.00 - 1.00 | 0.3722 |
| **Auto-HSCT** | 0.92 | 0.51 – 1.66 | 0.7851 |
| **Auto-HSCT during ICU stay** | 0.55 | 0.03 – 8.95 | 0.6751 |
| **Delay between auto-HSCT and ARDS** | 1.00 | 1.00 – 1.00 | 0.7933 |
| **Chemotherapy during ICU stay** | 0.73 | 0.53 – 1.00 | 0.0523 |
| **Delay between chemotherapy and ARDS** | 1.00 | 0.98 – 1.02 | 0.984 |
| **Duration of invasive mechanical ventilation** | 0.98 | 0.97 – 0.99 | <0.0001 |
| **Duration of non-invasive ventilation** | 0.98 | 0.95 – 1.01 | 0.1992 |
| **Duration of high flow nasal cannula** | 0.95 | 0.88 - 1.04 | 0.2543 |
| *ARDS : acute respiratory distress syndrome CI : confidence interval ECLS : extracorporeal life support HM : hematological malignancies HSCT : hematopoietic stem cell transplantation ICU : Intensive care unit LST : life-sustaining treatments* | | | |
